# Complexity of healthcare improvement – a comparative analysis of frameworks for improvement science

**DOI:** 10.3389/frhs.2026.1768259

**Published:** 2026-06-22

**Authors:** Caroline Ärleskog, Klas Gäre, Ann-Christine Andersson, Mattias Elg, Andreas Gremyr, Christina Petersson, Boel Andersson Gäre

**Affiliations:** 1 Department of Quality Improvement and Leadership, AKL, School of Health and Welfare, Jönköping University, Jönköping, Sweden; 2 Jönköping Academy for Improvement of Health and Welfare, Futurum, Region Jönköping County, Jönköping, Sweden; 3 Department of Management and Engineering, Linköping University, Linköping, Sweden; 4 Department of Psychotic Disorders, Sahlgrenska University Hospital, Göteborg, Sweden; 5 Center for Learning and Innovation, Region Jönköping County, Jönköping, Sweden

**Keywords:** complexity, frameworks, healthcare improvement, improvement science, social science

## Abstract

This study aims to critically compare and analyze relevant frameworks used in improvement science in order to examine how they address complexity as well as the contextual, social, and relational mechanisms that shape healthcare improvement. The study adopts a qualitative approach and is based on a comparative analysis of ten frameworks: KTA, CAIMeR, SHIFT, CAF, CAMM, CFIR, HPCM, IMQSE, NASSS and LHS. These frameworks were examined through a conceptual lens informed by theories of complexity as well as contextual, social, and relational mechanisms that shape healthcare improvement in practice. The findings reveal a fragmented conceptual landscape within improvement science, demonstrating substantial variation in how the frameworks address complexity and the contextual, social and relational mechanisms. A key observation is that most frameworks only partially and implicitly capture the complexity of healthcare systems, without clearly articulating how complexity influences improvement processes. This pattern is reflected in the overall results. One possible explanation is that many of the frameworks originate from fields such as Implementation Science and Health Informatics. However, this origin has implications when viewed from a Quality 3.0 perspective. Notably, Co-production in healthcare is largely absent from the analyzed frameworks. In addition, limited attention is given to the interactions between micro-, meso- and macro perspectives, which challenges the fundamental system perspective in healthcare improvement. In conclusion, the analyzed frameworks do not fully reflect the complexity of healthcare systems. Although many frameworks acknowledge contextual factors, fewer explicitly address the relational and social mechanisms through which healthcare improvement is enacted in practice.

## Introduction

Improvement science has emerged as an interdisciplinary field that seeks to provide conceptual and methodological rigor to the study and practice of healthcare improvement. The field has expanded rapidly and encompasses a wide range of theories, models and frameworks (1, 2). They all focus on concepts and their relationships but differ in depth and scope (3). In this study, we examine how conceptual frameworks that structure and shape the field of improvement science conceptualize complexity and related mechanisms in healthcare.

Healthcare improvement has evolved from approaches emphasizing standardization and compliance toward perspectives that recognize healthcare as relational, co-produced and context-dependent an era sometimes referred to as Quality 3.0 (4, 5). Increasingly, quality is understood as emerging from interactions among patients, professionals and organizations working towards shared aims (6). However, while earlier approaches are still relevant and have contributed significantly to advances in medicine, they are limited in addressing healthcare as a complex and dynamic system (7). In practice, healthcare improvement unfolds within rapidly changing and resource-constrained environments characterized by fragmentation, workforce pressures and shifting societal expectations (7–9).

Complexity has been frequently addressed in recent years (7, 8, 10–12), and refers to the fact that healthcare struggles with the unprecedented interacting challenges of demographic changes, the convergence of “health” and “care” needs and the disputes over who should pay for them, fragmentation of services, mismatches between workforce supply and system demand, a mushrooming of regulations and protocols, diminishing public trust in healthcare professionals, shrinking budgets etcetera (7). This evolving understanding of healthcare as a complex system characterized by non-linearity, interdependence, emergence and contextual variation, raises important questions about how improvement science conceptualizes and supports change. Frameworks that guide healthcare improvement must account for these dynamics. It therefore becomes essential to examine how influential frameworks in improvement science conceptualize complexity, including contextual, social and relational mechanisms.

### Rationale

Despite decades of effort, healthcare struggle to keep up and adapt to changes and challenges in ways that sustain quality, safety and value (13, 14). Many healthcare improvement initiatives also struggle to achieve sustained and wider impact. Evaluation of healthcare improvement initiatives often have a narrow focus on effectiveness and miss contextual factors which impedes the deeper learning and understanding of why initiatives fail (14). A recurring explanation is that initiatives often insufficiently account for the complex, contextual, social and relational nature of healthcare systems. Outcomes of healthcare improvement not only depend on technical design but also on how actors interact, interpret change, mobilize knowledge and co-produce solutions in specific organizational settings. Although complexity is widely acknowledged in the literature, it remains insufficiently operationalized in both science and practice (8). This is a gap where improvement science has a possibility to bridge between science and practice to “improve the improvement”.

Reed et al. (9) argues that even though the improvement community has grown considerably, there is no existing articulation of the scope of what matters to this community or how this aligns to the enquiries of improvement science. For example, interventions like education and training, which have important roles in quality and safety and are undertaken at vast scale, are often treated as undeserving of evaluation or research. Similarly, limited attention is directed to how healthcare professionals co-operate, co-produce and interact in various kinds of organizing [c.f. (14)] or to the need to span over sectors of co-producing actors and approach areas of both individuals and context [c.f. (15)]. Historically, quality and safety scholarships have been dominated by medical and safety sciences (16), while perspectives from social sciences have been comparatively underutilized or even neglected (15). As a result, there is a risk that social concepts of healthcare improvement are not consistently theorized and adapted, which challenges the bridge between improvement science and healthcare improvement in practice [c.f. (14)].

Since improvement science is diverse and inherently interdisciplinary, related frameworks vary in scope and orientation. This diversity enriches the field, but it also creates fragmentation (1, 14, 17) and the relationship between improvement science as theory and healthcare improvement as practice remains insufficiently articulated. It then remains unclear how frameworks conceptualize social concepts of healthcare improvement, particularly with respect to complexity and related contextual, social and relational mechanisms. Recent calls have urged further refinement and mapping of the improvement landscape, identified conceptual gaps and strengthening theoretical contributions (9). Taken together, these developments point to a need for greater conceptual clarity regarding how improvement science frameworks address complexity and related mechanisms. A comparative analysis of influential frameworks can therefore help illuminate strengths, blind spots and theoretical gaps in the current landscape.

#### Aim

The aim of this study is to critically compare and analyze relevant frameworks used in improvement science to examine how they address complexity and the contextual, social and relational mechanisms that shape healthcare improvement.

## A conceptual foundation for analysis

Healthcare improvement needs to be understood in relation to the nature of healthcare itself. Healthcare takes place across a spectrum of conditions, ranging from relatively standardized and predictable processes to situations marked by interdependence, contextual variation, adaptation and emergence (8, 10, 11). In this study, complexity is used as an analytical lens to understand improvement challenges that cannot be adequately captured through linear and mechanistic assumptions alone (7, 11, 18). We start from the observation that many contemporary improvement initiatives unfold in organizational settings where multiple actors and contextual conditions interact in dynamic ways, displaying features associated with Complex Adaptive Systems (CAS) (1, 8). This makes contextuality, together with social and relational mechanisms, central analytical concerns for improvement science rather than peripheral issues (7, 15).

### Contextuality and systems perspectives

Viewing healthcare improvement through a complexity lens foregrounds contextuality, referring to local circumstances in structures, policies and cultures as well as interdependencies between them (7, 8). Healthcare improvement is always embedded in local circumstances and linked to broader organizational and societal conditions. For this reason, healthcare improvement benefits from being understood through a systems perspective and across micro-, meso- and macro perspectives. The micro perspective concerns situated interactions in everyday practices; the meso perspective address organizational structures, teams and networks; and the macro perspective concerns policy, regulation and wider system conditions. These perspectives are analytically distinct but mutually shaping, which means that healthcare improvement is always contextual and multi-layered rather than universally transferable (7, 19). Systems perspectives challenge linear models that assume healthcare improvement can proceed without adequate consideration of context (7). They also draw attention to the fuzzy boundaries between contexts, meaning that they adapt, interact and co-evolve with each other (8).

### Social and relational mechanisms

Recognizing complexity also draws attention to the social and relational mechanisms through which healthcare improvement occurs (11), but which is rarely attributed to any central role within improvement science (15). From a social science perspective, healthcare can be understood in terms of relationships between social structures, networks and actors who interpret, negotiate, adapt or resist initiatives according to local logics and conditions (11, 20–22). Because healthcare is a service, healthcare improvement often involves co-productive processes in which patients may play an important role rather than being treated merely as passive recipients (4–6).

In light of this, organizing becomes crucial, referring to the coordination of interactions and actions (23). Sensemaking and sensegiving are important concepts in this regard. Sensemaking refers to how actors interpret ambiguity and make change meaningful enough to act on, while sensegiving refers to attempts to shape those interpretations (24–26). Healthcare improvement initiatives gain traction only when they are perceived as understandable, relevant and usable in practice (27–29). Their use is best understood in terms of integration into clinical activities and processes, including interactions in various contexts, coordination and communication activities and processes. In settings marked by uncertainty, interdependence and adaptation, rigid planning may be insufficient, making it important to support emergence, local adjustment and responses to feedback over time (30). Management also plays an important role in this context [c.f. (31)], and many myths about management are challenged when highlighting complexity. One of those myths is that healthcare is “failing” and that it could only be restored through hierarchical approaches, more administrative engineering or increased competition [c.f. (32, 33)]. This contrasts with the need for flexible structures and relationships capable of evolving as circumstances change, which is highlighted when acknowledging CAS characteristics in healthcare (7, 11, 34).

## Materials and methods

### Study design

The study adopts a qualitative approach [c.f. (35, 36, 65)], aiming to compare and analyze how some conceptual frameworks, used in improvement science, address the complexity and the contextual, social and relational mechanisms that shape healthcare improvement [c.f. (15, 37, 38)]. When conducting the study, we focused on frameworks that offer structured ways of understanding how healthcare improvement initiatives are designed, conducted and sustained in healthcare. The intention has not been to be comprehensive, but to compare a representative selection of relevant frameworks to enable comparisons, as well as to contrast the concepts within the analyzed frameworks with those in the described conceptual foundation for analysis.

### Identification strategy

For comparative analyses, identification was carried out in two steps: A) identifying concepts for the conceptual foundation and B) identifying frameworks of relevance for improvement science.

#### Identifying concepts for the conceptual foundation

To identify concepts for the conceptual foundation, a narrative approach has been applied, beginning with identification of relevant texts, those cited in introduction and rationale, in improvement science, and their references. The searches were performed in Google Scholar and PubMed based on terms found in the texts cited in introduction and rationale. The selected texts were critically analyzed in a process unfolded through multiple iterative cycles, each comprising [c.f. (27)]:

- conducting searches
- relevance assessment of search results
- review, reading and analysis of findings
- identification of categories, themes, and references to additional sources or documents
- refined searches based on updated parameters
- revisiting, verifying and re-reading documents, and continuous iteration as needed

Throughout the identification of concepts, detailed notes were maintained in separate documents (e.g., research logs, drafts). These notes were continuously shared with the author group for feedback, leading to revisions and the development of updated drafts. This cyclical approach ensured a thorough, reflexive and collaborative refinement of the analysis. The result was the concepts constituting the conceptual foundation: a) Complexity and CAS principles, b) Systems perspectives, c) Actors, structures and networks, d) Organizing, sensemaking and sensegiving, e) Use and adaptation, and f) Managing complex healthcare organizations.

#### Identifying frameworks for comparison and analysis

To identify frameworks, searches were conducted in Google Scholar and PubMed covering the period 1990–2025 using combinations of search words, such as healthcare, quality, improvement and framework. The initial searches produced a very large body of literature. The search process therefore followed an iterative strategy in which search strings were progressively refined. The results were screened for relevance to improvement science, including assessment of titles and abstracts based on the conceptual foundation, such as complexity, actors and sensemaking. The aim was not to produce an exhaustive inventory, but to identify some relevant frameworks for studies of healthcare improvement. The identified frameworks were then read in full-text to secure relevance, which was followed by a discussion within the author team. Through this process, ten frameworks widely referenced in improvement science were selected for comparison and detailed analysis.

Detailed notes were maintained in separate documents (such as research logs and drafts), followed by feedback from the author group, revisions, and successive drafts. The approach was intentionally inclusive to ensure that a broad range of potentially relevant articles advanced to a shortlist. These shortlisted articles were then subjected to a full-text review. The shortlist was subsequently assessed by co-authors, and this collaborative evaluation resulted in the selection of ten frameworks (Table 1).

**Table 1:** Selected frameworks in the first step of the analysi*s*.

Framework	Motivation
Knowledge To Action (KTA)	Chosen because of its focus on moving knowledge into action and the need for knowledge of healthcare improvement
Contexts, Actors, Interventions, Mechanisms and Results (CAIMeR)	Chosen due to the focus on how results in work practice arise from interventions and their contextual contingencies
Successful Healthcare Improvements From Translating evidence in complex systems (SHIFT)	Chosen because of the focus on interventions in CAS, characterized by uncertainty and surprise, which often defy intervention attempts
The Clinical Adoption Framework (CAF)	Chosen due to exhaustive components, which cover the most important parts of healthcare improvement and use
The Clinical Adoption Meta-Model is a temporal meta-model describing the clinical adoption (CAMM)	Chosen as it shows the vital importance of a time perspective in understanding adoption
The Consolidated Framework for Implementation Research (CFIR)	Chosen due to the descriptive dimensions of adoption, related to CAF
High Performing Clinical Microsystems (HPCM)	Chosen to cover important aspects in improvement of health and welfare services
The Integrated Map of Quality and Safety and e-Health (IMQSE)	Chosen because of its widespread application
The Nonadoption, Abandonment, and Challenges to the Scale-Up, Spread, and Sustainability of Healthcare Technologies (NASSS)	Chosen because it adds new perspectives in the concept of complexity and non-adaption and describes the dimensions in detail, while still also reflecting several parameters in the other frameworks
Learning Health Systems (LHS)	Chosen because of the focus on informatics, patient-clinician partnerships, incentives, continuous learning culture, that healthcare takes place in a culture of collaboration and adaptability, staff training and skill building to support learning processes

#### Analysis

In the first step, each framework was examined to identify main components in terms of origin and authorship, relevance to healthcare improvement, purpose (intent and focus areas), theoretical foundation, core components, mechanisms or processes of change and distinguishing features (Table 2). In the second step, the frameworks were analyzed in relation to how they address complexity and contextual, social and relational mechanisms that shape healthcare improvement. This step of the analysis was conducted based on the key concepts in the conceptual foundation of the analysis: Complexity and CAS principles (Table 3), Systems perspectives (Table 4), Actors, structures and networks (Table 5), Organizing, sensemaking and sensegiving (Table 6), Use and adaptation (Table 7) and Managing complex healthcare organizations (Table 8).

**Table 2:** Overview of framework*s*.

Framework, origin and authorship	Relevance	Purpose, intent and focus areas	Theoretical foundation	Core components	Mechanisms or processes of change	Distinguishing features
Knowledge To Action (KTA)	Healthcare builds on evidence, therefore knowledge translation and continuous education is relevant to healthcare improvement, which emphasis the learning, using learnings cycles like PDSA (Plan-Do-Study-Act)	Provides a structured approach to move knowledge into practice through two interconnected components: knowledge creation and the action cycle to implement evidence-based interventions, policies and guidelines	Based on insights from change theory, knowledge management, learning theory, behavioral change, and systems theory, forming a practical framework	Core concepts: Knowledge translation Continuing education Continuing professional development Knowledge transfer Knowledge exchangeResearch utilization Research utilization	Knowledge translation for continuing education in health professions includes needs to base it on the best available knowledge (evidence), the use of educational and other transfer strategies that are known to be effective, and the value of learning about planned action theories to better understand and influence change in practice settings	KTA is distinguished by its emphasis on learning and needs to base changes on evidence
Developed by Graham et al. (39) at the Canadian Institutes of Health Research						
Contexts, Actors, Interventions, Mechanisms and Results (CAIMeR)	CAIMeR is relevant due to the ability to create an understanding of why something works or does not work	Provides a conceptual framework and a theoretical model, which can help improve understanding of how and why social work functions in practice, not just whether it functions	Founded on a critical realism perspective on evaluation and explanation of social work practice. The theory has been developed through several years of empirical studies, together with social workers and in a critical manner	Core components: Context Actors Interactions Mechanisms Results	The framework pays attention to generative mechanisms in a micro-, meso- and macro-level in order to understand what is going on and to explain the results in a more qualified way. The framework enables a systematic analysis to identify what needs to be improved in a process and which mechanisms are significant in the situation	CAIMeR is distinguished by its basis in critical realism and its focus on questions of how and why. It emphasizes the importance of examining generative mechanisms across micro-, meso- and macro- levels
Developed by Blom and Morén (40) at Umeå University, Sweden.						
Successful Healthcare Improvement from Translating Evidence (SHIFT)	The guiding principles and the simple rules are considered necessary to achieve successful improvements. SHIFT can serve as a practical tool, offering simple rules to guide research and practice in evidence translation and improvement	Provides a practical tool to guide practice, as well as research, of evidence translation and improvement in CAS. It conceptualizes healthcare contexts as CAS characterized by unpredictability and uncertainty, which frequently challenge intervention efforts	SHIFT is empirically grounded and builds on existing work describing healthcare as CAS. SHIFT addresses a gap in literature considering implications of complexity in attempts to intervene and introduce evidence-based practices	SHIFT outlines three guiding principles: Act scientifically and pragmatically Embrace complexity Engage and empower	In addition to the three guiding principles, SHIFT contain 12 “simple rules” to navigate complexity and provide actionable guidance to practice and research	SHIFT stresses the importance of responding to unique conditions, managing unforeseen challenges and recognizing emergent effects of improvement activities. With its practical orientation and rule-based approach, SHIFT complements other frameworks that address actors across micro-, meso- and macro contexts
Developed by Reed et al. (1) at the National Institute of Health Research (NIHR), UK						
Clinical Adoption Framework (CAF)	CAF helps identify opportunities and obstacles for the successful implementation and use of Healthcare Information Systems (HIS). In doing so, CAF also aims to provide insights and contributions for further improvements	Provides a structured understanding of how staff/clinics adopt or refrain from adopting HIS	CAF builds upon theories and models from the disciplines of organization science, information systems and health informatics. It is an extension of the Benefits Evaluation (BE) framework and the Information System (IS) Success Model	CAF incorporates three conceptual perspectives of clinical adoption: Micro Meso Macro	At each level (micro, meso, macro) there is a feedback loop, where adoption efforts and results can reshape to higher levels	CAF distinguishes itself by viewing adoption as a dynamic process in which micro-, meso- and macro levels interact
Developed by Lau and Price (41) at the University of Victoria, Canada						
Clinical Adoption Meta-Model (CAMM)	CAMM aims to lead to healthcare improvement (technology is seen as a means, not an objective) and can be viewed as a framework for quality development through digitalization	Developed to support those studying, implementing and evaluating HIS	CAMM is based on traditional models for the adoption and implementation of ICT, as well as a socio-technical perspective that focuses on the relationship between technology, usage, organization, and results	CAMM describes four sequential dimensions of adoption: Availability Use Behavioral change Outcome change	CAMM identifies seven archetypical adoption trajectories, ranging from successful to failed implementations, which offer a structured way to analyze challenges in clinical system adoption	CAMM distinguishes itself through its focus on actual use and function, as well as by taking aspects of time into account
Developed by Price and Lau (42) at the School of Health Information Science, University of Victoria, Canada						
Consolidated Framework for Implementation Research (CFIR)	CFIR provides a pragmatic structure to assess factors shaping adoption and use, without prescribing fixed relationships between constructions, leaving these to be tested empirically	Provides a meta-framework of key concepts and domains that influence implementation	CFIR was developed by synthesizing existing models and frameworks. It builds on the comprehensive review of diffusion and dissemination theories by Greenhalgh et al. (43)	CFIR organizes constructions influencing implementation into five domains: The intervention The inner setting The outer setting Characteristics of individuals The implementation process	CFIR highlights the role of the users, implementers and influential stakeholders in determining outcomes	CFIR emphasizes the importance of context, both internal and external factors, which creates an understanding of why an intervention may work in one context but not another
Developed by Damschroder et al. (44) at Veterans Affairs (VA) Quality Enhancement Research Initiative (QUERI), USA						
High-Performing Clinical Microsystems (HPCM)	HPCM is widely recognized as a robust model for the design and evaluation of healthcare improvement. It offers both a conceptual and practical framework to simplify improvement efforts, contextualize performance and support organizational learning	Identify success factors in high-performance clinical microsystems and facilitate organizations in developing such systems, as well as creating a structure for continuous learning and improvement in healthcare	HPCM has an interdisciplinary scope and draws on quality criteria from the Malcolm Baldrige National Quality Award. The concept of clinical microsystems was first introduced by the Institute of Medicine (45, 46) and further developed by Dartmouth Institutes Microsystem Academy (47, 48)	HPCM identifies five key elements: Staff Patient Performance Leadership Information and IT	Each key element is linked to underlying principles that guide practice and inform evaluation of microsystem success	HPCM offers a structure to integrate different perspectives (micro, meso, macro) in healthcare improvement
Developed by Nelson et al. (47) at Darthmouth Institute for Health Policy and Clinical Practice, USA						
Integrated Map of Quality and Safety and e-Health (IMQSE)	IMQSE provides support in the planning and implementation of e-health solutions, considering both technology and the impact on healthcare quality and safety. IMQSE can also be used in evaluation and research to create a structure around what needs to be measured or investigated	Provides a conceptual framework that integrates quality and safety aspects with the application of E-Health	IMQSE takes core elements of quality and safety frameworks into account. It builds on a quality framework by Campbell et al. (49), that emphasizes five interdependent domains: effectiveness, efficiency, equity, acceptability and access. Effectiveness is considered central	IMQSE integrates three domains of e-Health applications with core elements of quality and safety frameworks. Recurring themes include, e.g., the characteristics of technology, processes, human factors in actors and their behaviors	Large potentials in use of information systems are identified for improvement of both quality and safety in healthcare. Interventions fail to live up to their potential when deployed in the real world due to lack of socio-technical aspects in design, implementation and use	IMQSE offers a structured conceptual map for evaluating e-Health interventions in terms of their contributions to safety and quality improvement
Developed by Car et al. (50) at Imperial College, London, UK						
Nonadoption, Abandonment and Challenges to Scale-up, Spread and Sustainability (NASSS)	By making complexity visible and tractable, NASSS helps practitioners design interventions that account for feedback, adaptation, and contextual variation. It highlights that healthcare improvement depends on the ongoing sensemaking and coordination of multiple actors across system levels	Explain why technology-supported health and care innovations are adopted, abandoned or fail to scale and sustain over time	Drawing on complexity science, actor–network theory, and diffusion of innovation, NASSS conceptualizes healthcare as a CAS in which outcomes emerge from interdependencies among technologies, people, organizations, and wider environments	The framework identifies seven interacting domains: Condition Technology Value proposition Adopter system Organization Wider system Embedding and adaptation over time	NASSS proposes that successful adoption occurs when complexity across these domains is recognized, aligned, and adaptively managed through learning and negotiation rather than controlled through linear planning	NASSS uniquely links theory and practice by operationalizing complexity itself as an analytic construct, offering a pragmatic tool for navigating uncertainty and co-producing sustainable innovation in healthcare systems
Developed by Greenhalgh et al. (27, 51) at the University of Oxford, UK						
Learning Health System (LHS)	The LHS embodies a comprehensive vision of healthcare improvement as a continuous, system-wide process of learning and adaptation. It promotes the integration of improvement, research, and implementation into daily practice, thereby linking data-driven insights into relational and organizational change	The LHS was conceived in the IOM-definition as a model for healthcare systems to continuously and systematically improve by linking science, informatics, incentives and culture. Its aim is to ensure that evidence is embedded in practice, patients are active partners and new knowledge is generated as an integral product of care	The LHS is grounded in systems thinking and improvement science, drawing on principles of CAS and organizational learning. It integrates the dual aims of knowledge generation (science) and care delivery (practice), emphasizing feedback and co-production as mechanisms of change	According to the IOM, a continuously learning health system aligns four interdependent domains: Science and informatics Patient–clinician partnerships Incentives Continuous learning culture	Learning occurs through iterative feedback loops that connect data, knowledge, and practice across all system levels. As defined by the IOM, care experiences are digitally captured to generate new knowledge, which informs improvement and innovation in real time. Subsequent work highlights that co-production (patients and professionals jointly designing, producing, and evaluating care) is central to making these learning cycles effective (51)	The LHS extends beyond traditional quality improvement by embedding feedback and co-production within the healthcare system itself. It treats learning as both a technical and social process—connecting evidence, interaction, and sensemaking in pursuit of better health for individuals and populations
The idea developed by the U.S. Institute of Medicine (IOM, now National Academies of Medicine) in 2007 (52) and later refined in Best Care at Lower Cost (53) has led to a multitude of LHS frameworks						

**Table 3:** How the frameworks capture complexity and incorporate CAS principle*s*.

Framework	Complexity and CAS principles
KTA	not in focus, but it addresses complexity implicitly through knowledge processes
CAIMeR	not in focus, but it addresses complexity implicitly through context mechanisms
SHIFT	focus on complexities in healthcare contexts, recognizing nonlinear interactions, unpredictability and dynamic change as inherent system features
CAF	not in focus, but it addresses the complexities of change and improvement
CAMM	not in focus, but it addresses complexity through its temporal framing of adoption stages
CFIR	not in focus, but it addresses a wide range of constructs that mirror the multidimensional nature of CAS
HPCM	not in focus, but it addresses contextual complexity implicitly by adopting a micro system perspective
IMQSE	not in focus, but it addresses socio-technical dimensions that align with CAS principles
NASSS	focus on CAS, emphasizing design, implementation and adaptation within complex contexts in a way that strongly reflects CAS principles, particularly through its focus on dynamic interactions and evolving system configurations
LHS	focus on CAS theory, making use of CAS terminology to highlight the need for feedback and addresses complexity by focusing on adaptability, continuous learning and strategies to moderate systematic challenges

**Table 4:** How the frameworks capture the micro-, meso- and macro perspectives and their interpla*y.*

Framework	Systems perspectives
KTA	not in focus
CAIMeR	In focus, it addresses generative mechanisms at different levels which is necessary to understand what is going on in a certain agency and explain the results in a more qualified way
SHIFT	in focus, addresses micro- (individuals) and macro perspectives
CAF	in focus, it stresses the interaction between individuals/groups (micro), organizational impact (meso) and external factors, including unintended social consequences (macro)
CAMM	not in focus
CFIR	in focus, contributes with a structured division of domains (e.g innovation, outer setting, inner setting and individuals) in a way that covers all three perspectives
HPCM	focus on micro perspective
IMQSE	not in focus, although identifying relevant sociotechnical aspects
NASSS	in focus, a wide perspective of organization, adopters, the wider context and embedding and long-term adaptation. A holistic view.
LHS	In focus, emphasizes strategies for adoption across all three levels

**Table 5:** How the frameworks address actors, structures and networks *.*

Framework	Actors, structures and networks
KTA	not in focus
CAIMeR	in focus, the theory is grounded in theories of social context and critical traditions, positioning actors and their interactions as central elements
SHIFT	not in focus, only implicitly as individuals are one perspective of the framework
CAF	in focus, identifying key actors like stakeholders, professionals, patients and families and highlighting their role in healthcare improvement
CAMM	not in focus
CFIR	in focus, engaging with identifying roles, culture, incentives, communication patterns and available resources—all of which are shaped by social structures
HPCM	In some focus, it identifies actors within the microsystem but pay no attention to e.g., networked relationships or processes of translation
IMQSE	in some focus, acknowledge key actors like professionals, patients and managers and highlights the importance of socio-technical interactions although not explicitly in terms of e.g., negotiation or network structures
NASSS	in focus, identifies adopters broadly like professionals, patients and carers, embedding them within wider system interactions
LHS	In focus, emphasizing collaboration and adaptability. Healthcare is considered as a collective learnings process, supported by relational structures rather than hierarchical directions

**Table 6:** How the frameworks address organizing, sensemaking and sensegiving*.*

Framework	Organizing, sensemaking and sensegiving
KTA	not in focus
CAIMeR	in focus, linking social mechanisms in social work practice to interactions where human intentions become externalized
SHIFT	in focus, conveys three principles (acting scientifically, embracing complexity, engaging and empowering) which implicitly support sensemaking in complex settings
CAF	in some focus, highlighting concepts of workflow integration, communication, training and education, all of which contribute to organizational sensemaking and structuring of work processes
CAMM	not in focus
CFIR	in focus, offering substantial content related to sensegiving, particularly within its process domain which e.g., includes learning needs, planning, engaging, assessing and adapting
HPCM	not in focus
IMQSE	not in focus, although highlighting the importance of human and socio-technical factors that influence adoption and failures
NASSS	in focus, identifying organizational capacity for innovation in leadership, processes, funding etc.—linking to broader interpretive and adaptive processes
LHS	in focus, emphasizing collaboration and adaptability as cultural foundations that support organizational learning and ongoing sensemaking

**Table 7:** How the frameworks emphasize the processes of use and adaptatio*n.*

Framework	Use and adaptation
KTA	in focus, emphasizing concepts like knowledge translation, continuing education, professional development, knowledge transfer, knowledge exchange and research utilization as central mechanisms for promoting use
CAIMeR	in focus, approaches adoption through social mechanisms in social work practice, framing human intentions as externalized within social interactions and highlighting the relational dynamics that shape outcomes
SHIFT	in focus, foregrounds individual engagement, dialogue, willingness and capability as essential factors for adoption, underscoring the role of active participation in facilitating change
CAF	in focus, placing use and user satisfaction at the core but do not explicitly examine actors' interaction, coordination or communication in the process of making use of innovations
CAMM	in focus, contributing with a temporal perspective on adoption, describing the order of conditions in availability of the innovation to use and change of behavior leading to outcomes
CFIR	in focus, identifies specific user roles, including leaders, deliverers and recipients. It incorporates mechanisms for learning and needs assessment, linking adopting to structured processes
HPCM	not in focus
IMQSE	not in focus, but touches on adoption within its socio-technical perspective
NASSS	in focus, explicitly addresses adaptation, adoption or non-adoption, emphasizing the dynamic interplay between innovations, adopters and context over time
LHS	not explicitly in focus, no focus on individual use or adoption (focus on culture and learning at a system level). It indirectly supports adoption and adaptation at an organizational level, through fostering a culture of continuous learning

**Table 8:** How the frameworks capture management and organizational development*.*

Framework	Managing complex healthcare organizations
KTA	not in focus
CAIMeR	not in focus
SHIFT	not in focus
CAF	in focus, offering a comprehensive set of change management components by incorporating governance, leadership, communication, training and education, workflow analysis and integration, stakeholder engagement, monitoring and evaluation
CAMM	not in focus, but it clarifies conditions under which adoption unfolds over time by organizing these into four stages of adoption
CFIR	in focus, identifying roles such as leaders, facilitators and support staff as well as their needs, capabilities, and motivations
HPCM	sin focus, providing detailed descriptions of leadership within the microsystem and highlighting the importance of purpose, clear goals, positive vulture and advocacy within the larger organization. It emphasizes recognition, information sharing and legitimization as core managerial practices.
IMQSE	not in focus, but it addresses several key issues such as innovation processes, user attitudes, communication, project management, effective leadership and evaluation. Recognizing the need for more attention to socio-technical dimensions in designing, developing and deploying applications in order to avoid failures and promote organizational success
NASSS	focus on managing complex healthcare organizations, addressing several concepts associated with management such as leadership, processes, funding and organizational capacity to innovate
LHS	focus on managing complex healthcare organizations, aligning management with network-based organizational architecture to facilitate the improvement of current healthcare. It emphasizes co-production, continuous learning and understanding complexity as essential

## Results

This section initially provides a comparing overview (Table 2) of the main components of each framework, based on the following aspects:

- Origin and authorship
- Relevance to healthcare improvement
- Purpose—intent and focus areas
- Theoretical foundation
- Core components
- Mechanisms or processes of change
- Distinguishing features

### Mapping the frameworks against the conceptual foundation

Tables 3–8 presents overviews of the comparisons and analysis of the frameworks, all parts of structuring and shaping the field of improvement science, in relation to the following key concepts:

a) Complexity and CAS principles (Table 3)
b) Systems perspectives (Table 4)
c) Actors, structures and networks (Table 5)
d) Organizing, sensemaking and sensegiving (Table 6)
e) Use and adaptation (Table 7)
f) Managing complex healthcare organizations (Table 8)

#### Complexity and CAS principles

To understand the essence of complexity and its implications is important for the understanding of success or failure in healthcare improvement. The results show how the frameworks differ considerably in the degree to which they engage with complexity and incorporate CAS principles (Table 3). Most of the frameworks implicitly touch on complexity, but only a handful explicitly address CAS principles.

#### Contextuality and systems perspectives

The application of micro-, meso- and macro perspectives provides an important analytical lens for understanding healthcare improvement. These levels capture the interplay between individual users, organizational structures and broader societal contexts. Table 4 illustrates how the frameworks relate to the three perspectives, as well as the interplay between them.

#### Social and relational mechanisms

##### Actors, structures and networks

Actors in organizing and managing healthcare improvement in complex healthcare organizations call for an understanding of healthcare improvement as situated within social networks and structures. This highlights the importance of actors' interactions, negotiations and translations of meaning across professional and patient groups. The degree to which frameworks address actors, networks and processes of translation and negotiation varies widely, from a great extent to not at all (Table 5).

##### Organizing, sensemaking and sensegiving

Furthermore, the organizing of healthcare improvement can be understood through concepts such as sensemaking and sensegiving. Similar to previously presented social concepts, this concept also receives uneven attention across the frameworks (Table 6).

##### Use and adaptation

The analyzed frameworks also vary in how they address the processes of use and adaptation of knowledge and healthcare improvement. Most frameworks address these concepts in one way or another, albeit to varying degrees. Only a few emphasize the processes of use and adaptation to any significant degree (Table 7).

##### Managing complex healthcare organizations

Finally, managing complex healthcare organizations requires collaboration, distributed leadership and skills that extend beyond top-down perspectives. This recognizes that healthcare is broader than the treatment of disease alone. Management considerations in complex healthcare organizations vary considerably across the frameworks (Table 8). Some frameworks focus explicitly on this concept, some relate to it implicitly, while others do not address it at all.

## Discussion

The comparison and analysis of the frameworks highlight a fragmented conceptual landscape within improvement science. A central observation is that most frameworks only partially capture the complexity of healthcare, mostly implicitly and without articulating how complexity shapes healthcare improvement. As healthcare improvement occurs in systems characterized by interdependence, emergence and unpredictability (10, 11), limited engagement with CAS-principles may constrain the ability of some frameworks to account for these dynamics. The limited engagement with complexity reflects in how the frameworks address social concepts of healthcare improvement. As an example, few frameworks address management nor attempt to capture actors in networks and structures. This constrains the ability to capture the relational processes through which much healthcare improvement arises, including collaboration, negotiation and translation across roles, professions and settings (20, 54). Because of this, many healthcare improvement initiatives cannot rely solely on top-down control and may require collaborative leadership, distributed decision-making and an understanding for interdependence (7, 8, 55).

This pattern may partly reflect the intellectual roots of these frameworks. Many frameworks originate from implementation science, early improvement science or health informatics, traditions that follow more linear logics and often privilege structured models of interventions and adoption, in line with the logic of clinical research. Such approaches tend to emphasize factors influencing implementation rather than the dynamic social processes through which actors interpret, negotiate and enact change in practice. Frameworks that are influenced by implementation science may, for example, describe contextual mechanisms but they rarely consider how interventions themselves reshape the context or how learning unfolds over time. Compared to improvement science, there are several notable distinctions. Some relate to the consideration of the temporal dimension [c.f. (9)], others lie in improvement science emphasizing continuous learning, iteration and capability building within the system to achieve enhanced quality of care. Influences from implementation science may, for example, explain why organizing, sensemaking and sensegiving are underdeveloped concepts in most frameworks. In CAF, for example, focus is directed towards adoption in terms of use and user satisfaction, but there are no cues given to why use occurs for understanding adoption. There are no references to concepts or synonyms for sensemaking or sensegiving, even if sensemaking is vital in users′ adoption of healthcare improvement.

Another central observation refers to co-production. Co-productive aspects of healthcare improvement are only weakly reflected in most of the frameworks. This is noteworthy given the growing emphasis on patient participation, value creation and co-production in contemporary healthcare (6), as well as co-production being highlighted in the Quality 3.0 perspective for the same reason (4, 5). Even though co-production is essential, there is no standardized recipe for what works, when or why (54, 56, 57). Improvement science could help us increase our understanding of co-production, yet this goes under the radar in the frameworks. Looking at HPCM, as an example, the patient perspective is highlighted as important but is only considered from the professionals' perspective and not from the patient's own perspective and lived experience. The absence of co-production is even more notable as the micro perspective is rather well covered by the frameworks, unlike the meso- and macro perspectives. From a system perspective, it is possible to state that only a handful of frameworks incorporate all three perspectives and emphasize their interplay. This contrasts with the systems thinking that is central to healthcare improvement (4, 5). Something of a catch 22-situation arises as this limits the understanding of the bigger picture (29), further affecting the possibilities of capturing the complexity of healthcare improvement (10, 11, 27).

### Implications for science, practice and future research

#### Implications for improvement science

Improvement science has expanded rapidly in recent years (2), prompting reflection on how it bridges to healthcare improvement in practice. This expansion has contributed to a fragmented conceptual landscape, which paves way for an incoherent understanding of healthcare improvement. Understanding the dynamic of healthcare improvement requires a fundamental grasp of its nature and objectives and therefore benefits from multidisciplinary perspectives. The current diversity within improvement science (8, 29, 58) enriches the field by introducing a range of perspectives, yet it also contributes to conceptual fragmentation (9). This study contributes to strengthening the coherence between theory and practice [c.f. (14)] by examining how the frameworks relate to complexity and to contextual, social and relational mechanisms. In doing so, the study may support more informed and reflective selection of frameworks and theoretical concepts when studying healthcare improvement. At the same time, the results also point to a need for continued theoretical development that extends beyond linear thinking, technical change and clinical knowledge. Pursuing a comprehensive “grand theory” [c.f. (59)] would be unrealistic, as such generalizations rarely translate effectively to specific contexts of practice (60). Nevertheless, future theoretical development may benefit from more explicitly addressing complexity as well as contextual, social and relational mechanisms. Drawing more on insights from the social sciences could then strengthen both the explanatory capacity and the practical relevance of frameworks within improvement science.

#### Implications for healthcare improvement

This study suggests that healthcare improvement requires competencies that extend beyond clinical expertise, just like Batalden and Stoltz (61) pointed out already in 1993. Improvement initiatives must therefore attend not only to technical implementation but also to how professionals and patients interpret these initiatives, experience their usefulness and integrate them into everyday practice. Education and training in healthcare improvement, grounded in improvement science, should consequently emphasize usability, sensemaking and co-production within complex healthcare organizations, alongside technical methods and tools (62, 65, 66). The results of this study may also support navigation among the various frameworks within improvement science by highlighting their respective areas of emphasis as well as their blind spots. In doing so, the study may help mitigate the fragmentation that has emerged from the field's diversity [c.f. (1, 14, 17)] by clarifying how different frameworks address social concepts in healthcare improvement. In this way, the study contributes to making improvement science more accessible, which is important given that the role of theory has historically been underrecognized in healthcare improvement. By “demystifying” theory [c.f. (60)], the study may help strengthen improvement initiatives by grounding them more firmly in improvement science. A stronger theoretical foundation may also enhance the reproducibility and overall impact of improvement initiatives.

#### Directions for future research

The results indicate a need for more empirical research linked to the presented frameworks in combination with a theoretical foundation. Future studies should examine how frameworks and theories from the conceptual foundation in improvement science function in practical settings, how they are adapted over time and how they interact with local cultures, histories and path dependencies. Such research could support the development of more context-sensitive and transferable approaches to healthcare improvement, in line with the, in improvement science, central questions of what works for whom, when, where and how (14). Empirical research using the analyzed frameworks and others in combination with the conceptual foundation could strengthen the alignment between theory and practice, thus advancing both improvement science and achieving sustainable healthcare improvement.

### Methodological reflections

This study adopted a pragmatic and deliberately broad approach, prioritizing conceptual openness, relevant to improvement science. We are aware that there are more frameworks, which in another setting, could have been included. However, the study contributes to sensitizing concepts relevant to improvement science and the area of usefulness. Sensitizing concepts are relevant and demonstrate the distinction between a framework (improvement science) and its application (healthcare improvement). In this mutual dependency of improvement science and healthcare improvement, both aiming to reach and understand useful improvement (14), we argue that this study can be a useful complement.

The chosen approach enabled the integration of social science perspectives on complexity, which are often underrepresented in improvement science but have significant implications and relevance as they advance our understanding and address gaps in the existing body of knowledge (15, 16) The pragmatic approach, rigor and transparency have been carefully handled to maintain trustworthiness (63) and usefulness. To strengthen the rigor of the process and interpretations, the emerging results were continuously discussed and agreed upon among all authors, all of whom have extensive experience in healthcare improvement and improvement science. Transferability in the pragmatic approach is mainly about usefulness, which relates to the understanding of dynamic processes and practices in [complex] organizations (64), aiming at developing tools for solving real-world problems.

We are convinced that the comparative analyses offer a meaningful contribution by synthesizing and problematizing some existing frameworks' usefulness from a complex system perspective. Thus, the comparative analysis is a meaningful contribution that helps “to refine and advance mapping the improvement landscape by identifying gaps and increasing contributions from diverse perspectives” [(9):1], thereby responding to both academic and societal needs.

## Conclusion

The main conclusion is that the frameworks do not fully reflect the complexity of healthcare improvement. While many frameworks acknowledge contextual factors, fewer explicitly address the relational and social mechanisms through which healthcare improvement is enacted in practice.

## Data Availability

The original contributions presented in the study are included in the article/Supplementary Material, further inquiries can be directed to the corresponding author.
